# An acute bout of localized resistance exercise can rapidly improve inhibitory control

**DOI:** 10.1371/journal.pone.0184075

**Published:** 2017-09-06

**Authors:** Hayato Tsukamoto, Tadashi Suga, Saki Takenaka, Tatsuya Takeuchi, Daichi Tanaka, Takafumi Hamaoka, Takeshi Hashimoto, Tadao Isaka

**Affiliations:** 1 Research Organization of Science and Technology, Ritsumeikan University, Shiga, Japan; 2 Graduate School of Sport and Health Science, Ritsumeikan University, Shiga, Japan; 3 School of Medicine, Tokyo Medical University, Tokyo, Japan; University of Akron, UNITED STATES

## Abstract

The positive effect of acute resistance exercise on executive function, such as inhibitory control (IC), is poorly understood. Several previous studies have demonstrated this effect using whole-body resistance exercise. However, it remains unclear whether localized resistance exercise performed using only limited muscle groups could also acutely improve IC. Thus, the present study examined the effect of an acute bout of localized resistance exercise on IC. Twelve healthy men performed a color-word Stroop task (CWST) before and immediately after the experimental conditions, which consisted of 2 resistance exercises and a resting control (CON). Bilateral knee extension was used to create 2 resistance exercise conditions: light-intensity resistance exercise (LRE) and high-intensity resistance exercise (HRE) conditions, which were 40% and 80% of one-repetition maximum, respectively. The resistance exercise session was programmed for 6 sets with 10 repetitions per set. The CWST-measured IC was significantly improved immediately after both LRE and HRE, but it did not improve immediately after CON. However, the improved IC was significantly greater in HRE than in LRE. The present findings showed that IC could be rapidly improved by an acute bout of localized resistance exercise, especially with high-intensity. Therefore, we suggest that in addition to whole-body resistance exercise, localized resistance exercise performed using limited muscle groups may be sufficient for improving IC.

## Introduction

Executive function (EF) involves three aspects: shifting, updating, and inhibition [1]. EF is known to be chronically impaired by aging [2] and by various chronic diseases, such as Alzheimer’s disease [3], type 2 diabetes mellitus [4], and chronic obstructive pulmonary disease [5]. Moreover, impaired EF is associated with poor prognosis in older people [6, 7], especially sedentary individuals [7].

Previous studies have reported that an acute bout of aerobic exercise can improve several aspects of EF, especially inhibitory control (IC), in various populations [8–15]. Moreover, previous studies demonstrated that aerobic exercise-induced improvement of IC was related to enhanced neural activity in the brain [8–10]. Furthermore, Byun et al. demonstrated that the aerobic exercise-induced enhancement of cerebral neural activity was related to increased arousal. Based on these findings, previous studies have proposed that the potential mechanism underlying the improvement in IC is associated with enhanced cerebral neuronal activation and arousal [8–10].

Compared to what is known about the effects of aerobic exercise, it is poorly understood whether an acute bout of resistance exercise would also have a positive effect on IC and its mechanisms. Previous studies showed that whole-body resistance exercise involving multiple events (e.g., combinations of leg press, leg extension, bench press, and biceps curl, etc.) could acutely improve IC [16,17]. Whole-body resistance exercise is performed by recruiting a large number of muscle groups during an exercise session. The same recruitment of a large number of muscle groups is also observed in aerobic exercise because multiple joints (e.g., ankle, knee, and hip joints) are used [18]. In contrast, localized resistance exercise (e.g., knee extension only), especially in the case of single-joint resistance exercise, is performed by recruiting a limited number of muscle groups. Thus, compared to aerobic exercise and whole-body resistance exercise, determining whether localized resistance exercise could improve IC could help understand the effect of limited muscle stimulation. In addition, localized resistance exercise has been widely employed to examine various effects (e.g., biological and physiological responses of the skeletal muscles) of resistance exercise [19,20]. Taken together, clarifying the effect of localized resistance exercise on IC improvements may be useful for determining the mechanism underlying the improvements in IC, such as brain-skeletal muscle network [14,21–23]. However, to the best of our knowledge, the effect of localized resistance exercise on IC remains unclear. Previous studies have demonstrated that localized resistance exercise could enhance cerebral neural activation and arousal [24,25]. Based on these findings, we hypothesized that, similar to the effects of whole-body resistance exercise, localized resistance exercise would acutely improve IC.

A meta-analysis by Chang et al. (2012) showed that improved cognitive function immediately after aerobic exercise was greater for lighter-intensity exercise than for higher-intensity exercise [11]. Their findings suggest that high-intensity exercise might attenuate IC improvements immediately after exercise. Furthermore, Chang and Etnier (2009) reported that improved IC immediately after whole-body resistance exercise was greater for moderate-intensity exercise than for low-intensity exercise or high-intensity exercise, and the effects were comparable between low-intensity exercise and high-intensity exercise [16]. Therefore, it is speculated that improved IC immediately after localized resistance exercise would be lower for high-intensity exercise than for light-intensity exercise. However, a recent study by Chang et al. (2015) demonstrated that improved IC immediately after aerobic exercise was greater following exercise of a moderate duration (i.e., 20 min) than following exercise of a longer duration (i.e., 45 min) [26]. Importantly, because of the large number of exercise events, whole-body resistance exercise requires a relatively longer duration (i.e., > 20 min) than standard localized resistance exercise [27]. Thus, the relatively small benefit of high-intensity whole-body resistance exercise for IC improvements may be associated with the relatively long exercise duration. Based on these findings, we hypothesized that IC improvement immediately after standard localized resistance exercise with a short duration (i.e., about 20 min) would be greater following high-intensity exercise than following light-intensity exercise.

To test our hypotheses, we examined whether an acute bout of localized resistance exercise using only knee extension would rapidly improve IC. In addition, to determine the effect of exercise intensity on IC improvement immediately after exercise, we compared the magnitude of the IC improvement between light-intensity resistance exercise (LRE) and high-intensity resistance exercise (HRE).

## Methods

### Ethics statement

The investigation was conducted according to the principles expressed in the *Declaration of Helsinki*. All procedures in this study were approved by the Ethics Committee of Ritsumeikan University (IRB-BKC-2014-027). The subjects were informed of the experimental procedures and potential risks and provided written consent to participate in this study.

### Subjects

Twelve healthy male young subjects (mean ± SD, age: 22.9 ± 1.4 years, height: 171.3 ± 5.3 cm, weight: 67.3 ± 10.2 kg) participated in this study. All subjects were right-hand dominant and free of any known neurological, cardiovascular, and pulmonary disorders, as well as free from color-blindness, and abnormal vision. The subjects were instructed to avoid strenuous physical activity in the 24 h prior to each experimental session. Each subject also abstained from food (overnight fasting), caffeine, and alcohol for 12 h prior to each experiment.

### Experimental conditions

**Fig 1:**
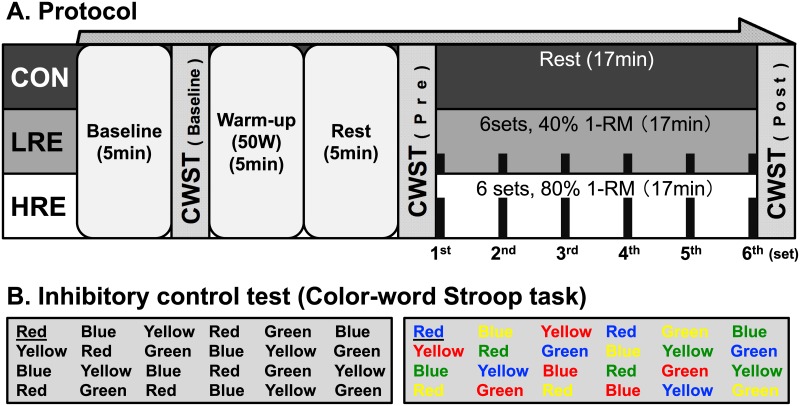
Experimental design and typical examples of the color-word Stroop task (CWST) measurement. Panel A shows the time line of the experimental session. To assess inhibitory control (IC), the CWST was measured at three time points: baseline, before (i.e., Pre) and immediately after (i.e., Post) the experimental session. The control (CON) condition consisted of the subject being at rest in a sitting position. The light-intensity resistance exercise (LRE) and high-intensity resistance exercise (HRE) conditions were applied to 40% and 80% of the one-repetition maximum (1-RM), respectively. Both exercise conditions were programmed for 6 sets (displayed using black bars) with 10 repetitions per set. The rest intervals between sets lasted 3 min. The total experimental time was at 17 min. Panel B shows typical examples of neutral and incongurent trials from the CWST. The neutral trial displays the color names presented in black text (e.g., when the word “RED” was printed in “black” ink, correct answer is “RED”). The incongruent trial displays the color names presented in a different-colored text (e.g., when the word “RED” was printed in “blue” ink, correct answer is “RED”).

The experimental conditions and design are shown in Fig 1A. The main experiment consisted of 3 experimental conditions; there were 2 resistance exercise conditions and a resting control (CON) condition. The 2 resistance exercise conditions used bilateral knee extension to produce LRE and HRE, which were 40% and 80% of one-repetition maximum (1-RM), respectively. The bilateral knee extension exercise was performed using an exercise machine (Life Fitness, IL, USA). The resistance exercise protocol was programmed for 6 sets with 10 repetitions (1-s concentric contraction/1-s eccentric contraction) per set. The rest intervals between sets lasted 3 min. The total experimental time was 17 min. All subjects completed the LRE and HRE conditions, but some subjects lowered the exercise load during HRE to maintain proper form. The decrease exercise loads required for the subjects to complete the HRE were performed using a range of 1–5 kg. This information has added the “1-RM” subsection in “Measurement” of Methods. The CON condition consisted of being at rest in a sitting position on the knee extension exercise machine for the same length of time (i.e., 17 min) as the exercise conditions.

### Measurements

#### CWST-measured IC

To assess IC, in the present study, we used the color-word Stroop task (CWST). CWST is a well-known paradigm for investigating aspects of a higher cognition that depend on EF [28]. Specifically, CWST focuses on selective attention to specific information and the IC of a prepotent response during decision-making tasks involving stimuli and responses [1,29]. The CWST in the present study was programmed by modifying an Excel Visual Basic for Applications, as described in our previous studies [12–15]. We measured both reaction time and response accuracy using the CWST as previously described [12–15]. The following instructions were given to the subjects: “you must perform as accurately and quickly as possible.” The stimulus words were four color-names (“RED”, “YELLOW”, “GREEN” and “BLUE”), and they were presented on a 98-inch display. All words were written in Japanese. We prepared a color-labeled ten key board: number 1 key was labeled red, number 2 key was labeled yellow, number 3 key was labeled green, and number 4 key was labeled blue. The subjects were required to press the color-labeled key that corresponds to the text meaning (i.e., matching response).

Typical examples of the CWST measurement are presented in Fig 1B. A reverse-Stroop interference score is evaluated from the difference between reaction times for word-reading and incongruent word-reading trials [30]. To assess IC, this score is more appropriate for calculating the interference in this response modality than that of the Stroop interference score, which is evaluated from the difference between response times of color-naming and incongruent color-naming trials [30]. Thus, to calculate a reverse-Stroop interference score, the present study employed two types of CWST, which consisted of the word-reading (i.e., neutral trial) and incongruent word-reading (i.e., incongruent trial) trials. The neutral trial, which is a control task, displayed the color names presented in black text. The incongruent trial, which is an interference task, displayed the color names presented in a different-colored text. The words for each type of CWST were presented in a random order. Each trial consisted of 24 stimulus words and was repeated for three trials. A reverse-Stroop interference score was calculated as (reaction time of incongruent trial—reaction time of neutral trial) / reaction time of neutral trial × 100 [13–15,30].

#### 1-RM

Prior to the main experiment, the subject’s 1-RM was determined by the successful lift of the bilateral knee extension exercise on the first visit day. The 1-RM value was used to determine the loads for the LRE and HRE conditions. The measurement procedure for 1-RM is described in our previous paper [31]. Briefly, the 1-RM trial was designed using increments of 10 kg until 60–80% of the perceived maximum is achieved. Then, the load was gradually increased by 1–5 kg weights until lift fail, in which the subject was not able to maintain proper form or to completely lift the weight. The last acceptable lift with the highest possible load was defined as 1-RM. The mean value of the subjects’ 1-RM was 116.9 ± 3.4 kg.

#### Cardiovascular responses

The evaluation of cardiovascular response was performed using heart rate (HR) and mean arterial blood pressure (MAP). HR was monitored continuously via telemetry (RS 400; Polar Electro Japan, Tokyo, Japan). Systolic blood pressure (SBP) and diastolic blood pressure (DBP) were measured via a mercury manometer. MAP was calculated as (SBP − DBP) / 3 + DBP.

#### Skeletal muscle responses

**EMG activity**—The evaluation of skeletal muscle response was performed using electromyographic (EMG) activity. Prior to the application of electrodes, the subject’s skin was shaved, abraded, and cleaned with alcohol to minimize skin impedance. EMG signals were recorded with surface electrodes from the quadriceps vastus lateralis (VL), vastus medialis (VM), and rectus femoris (RF) muscles. The EMG signals were amplified 1000 times, band-pass filtered between 10 Hz and 500 Hz, and sampled at 1000 Hz (MQ-Air; Kissei Comtech, Nagano, Japan). The EMG activity of each muscle during the exercise conditions was quantified as the integration of the rectified EMG (iEMG) over 1 s. Thereafter, the EMG data were normalized to the highest EMG (the average value over 1 s) that was obtained during the 3 trials of maximal voluntary contractions (MVC), which was measured by establishing a sufficient rest interval (approximately 10 min) after the experimental session was completed. The EMG activities of the quadriceps muscle were represented as the average from all 10 repetitions in every set.

**NIRS-derived deoxygenation**—Local oxygenation profiles in the quadriceps VL muscle during the experimental exercises were measured using near-infrared spectroscopy (NIRS) (NIRO 200; Hamamatsu Photonics, Shizuoka, Japan). Changes in deoxy-hemoglobin/myoglobin (Hb/Mb), oxy-Hb/Mb, and total-Hb/Mb were sampled at 5 Hz and then averaged at 1 s. The present study predominantly analyzed the changes of deoxy-Hb/Mb, which is a marker of muscle deoxygenation. The deoxy-Hb/Mb-derived muscle deoxygenation has been employed as a reliable estimator of changes in intramuscular oxygenation states during exercise. The values of deoxy-Hb/Mb were calibrated using an arterial occlusion method for 10 min [32], with an ischemic pressure for 300 mmHg. The arterial occlusion began 5 min after the MVC trials finished. During arterial occlusion, a maximal plateau value of deoxy-Hb/Mb was obtained for calibration. The baseline value for calibration was defined as the mean value measured over 60 s during resting before the onset of the experimental exercise. The highest value of deoxy-Hb/Mb during every set was expressed as a percentage using baseline and maximal plateau values.

#### Psychological responses

**RPE**—The rating of perceived exertion (RPE) was measured to assess the effort expended during exercise. This scale ranges from 6 (no exertion) to 20 (maximal exertion) [33].

**FAS**—The felt arousal scale (FAS) was measured to assess arousal level, and this scale ranges from 1 (low arousal) to 6 (high arousal). For example, high arousal level is "excitement", and low arousal level is "relaxation" [34].

**VAS**—The visual analog scale (VAS) for the CWST consisted of questions of three psychological types that assess mental fatigue, the ability to concentrate, and motivation. Each VAS was labeled from 0 mm (i.e., not at all) to 100 mm (i.e., extremely). The subjects drew lines to indicate their response [13–15].

### Experimental design

On the first visit, subjects were familiarized with the IC test using the CWST [28]. Their 1-RM was then measured for the main experimental equipment. To reduce any learning effects, the CWST was practiced at least 10 times for both CWST trials until the subject achieved a constant score [12], defined by an average reaction time over 5 practice sessions for each trial that was under 500 msec. The 1-RM was determined by a successful knee extension contraction. Thereafter, subjects came to the laboratory on 3 additional occasions to participate in the main experiment. The order of the experimental conditions was randomly assigned and counterbalanced. Each condition was separated by at least 72 h. All experiments were performed at the same time (± 1 h) and started between 9:00AM and 11:00AM.

On the experiment days, the subject practiced the CWST prior to the measurement of baseline data to avoid any learning effect. The subject then rested for 5 min and underwent measurement of their baseline psychological and cardiovascular parameters. In the baseline data, HR and MAP were measured. Additionally, fasting fingertip blood glucose level on the day of the experiment was measured using a glucose analyzer (Glutest Neo Alpha; Sanwa Kagaku Kenkyusho, Nagoya, Japan) to assess the fasting state, and there were no significant differences between the 3 experimental days (CON: 93.3 ± 9.0, LRE: 95.2 ± 7.8, HRE: 93.0 ± 8.0 mg/dl). After baseline data were recorded, the subject performed the baseline CWST in a sitting position on the knee extension machine.

Next, the subject performed a warm-up exercise at 50 W for 5 min using a bicycle ergometer (Life Fitness, IL, USA). In the warm-up exercise, HR and RPE were recorded. The values of HR (CON: 92.3 ± 9.3, LRE: 91.4 ± 10.1, HRE: 94.0 ± 7.9 bpm) and RPE (CON: 8.2 ± 1.5, LRE: 8.0 ± 1.6, HRE: 8.1 ± 1.8) did not differ significantly between the 3 experimental days. After the warm-up, the subject rested for 5 min, and pre-exercise CWST was then measured. The results of the CWST did not differ significantly between the baseline and pre-intervention data for any of the 3 conditions.

Subsequently, each experiment condition began. Following completion of the experimental condition, post-exercise CWST was measured. This measurement of CWST was concluded within 5 min after completion of the experiment in all trials of the present study. During the experimental session, HR and MAP were measured at every set in the 2 exercise conditions. The CON condition was measured at the same experimental time as the exercise conditions. The RPE was measured at every set in the exercise conditions. The EMG activity and NIRS-derived deoxygenation in the skeletal muscle were measured over the experimental session in only the exercise conditions. To assess the psychological conditions for CWST, FAS and VAS were utilized after measurements of CWST in all conditions.

### Statistical analysis

The data are expressed as the mean ± SD. The HR, MAP, RPE, iEMG, NIRS-derived deoxy-Hb/Mb were analyzed by using a two-way (set × condition) repeated-measures analysis of variance (ANOVA). Moreover, the CWST-measured reaction time and response accuracy were analyzed by using a three-way (type of CWST × pre/post × condition) repeated-measures ANOVA. Moreover, CWST-measured interference score was analyzed by using a two-way (pre/post × condition) repeated-measures ANOVA. In addition, the changes in psychological parameters, which are also calculated by post—pre, were analyzed by using a one-way (only the condition) repeated-measures ANOVA. These ANOVAs were performed for all analyses because the data conformed to normal distributions. If the sphericity assumption was not met, Greenhouse-Geisser corrections were used. Partial eta squared (*η*_p_^2^) values were determined as a measure of the effect size for all the main effects and interactions. Specific differences were identified with a Bonferroni *post-hoc* test. The statistical significance level was defined at *P* < 0.05. In addition, Cohen’s *d* effect sizes using the means and pooled standard deviations were calculated, along with 95% confidence interval, to determine the magnitude of differences in outcome variables between conditions. The strength of effect sizes was interpreted as trivial (0 ≤ *d* ≤ 0.19), small (0.20 ≤ *d* ≤ 0.49), medium (0.50 ≤ *d* ≤ 0.79), and large (0.80 ≤ *d*) [35]. All statistical analyses were conducted using IBM SPSS software (version 19.0; International Business Machines Corp, NY, USA).

## Results

### Baseline data

The reaction times and response accuracies for neutral and incongruent trials of the CWST during three conditions at baseline are shown in Table 1. There were no significant differences among all three conditions. Additionally, HR (CON: 68.0 ± 8.8, LRE: 68.1 ± 11.1, HRE: 68.5 ± 8.7 bpm) and MAP (CON: 89.7 ± 7.9, LRE: 87.3 ± 9.7, HRE: 87.8 ± 7.7 mmHg) before the CWST did not differ significantly among the three conditions.

**Table 1:** Baseline data for the results of the color-word Stroop task (CWST).

	Reaction time (msec)	Response accuracy (%)
***Neutral task***		
**CON**	10940 ± 1849	98.3 ± 1.3
**LRE**	10942 ± 1352	98.6 ± 1.3
**HRE**	10974 ± 1745	97.7 ± 2.5
***Incongruent task***		
**CON**	12176 ± 2055	98.4 ± 1.7
**LRE**	11971 ± 1538	98.5 ± 1.7
**HRE**	12279 ± 2181	98.7 ± 1.7

Values are presented as means ± SD. CON; control, LRE; low-intensity resistance exercise, HRE; high-intensity resistance exercise.

### Physiological and psychological responses during exercise

The cardiovascular responses in all conditions are shown in Table 2. The values of HR and MAP in the pre-intervention data were similar among all conditions. There were significant main effects for set (HR: *F*_6, 66_ = 218.17, *P* < 0.01, *η*_p_^2^ = 0.95; MAP: *F*_6, 66_ = 18.60, *P* < 0.01, *η*_p_^2^ = 0.63), condition (HR: *F*_2, 22_ = 210.60, *P* < 0.01, *η*_p_^2^ = 0.95; MAP: *F*_2, 22_ = 18.46, *P* < 0.01, *η*_p_^2^ = 0.63), and set × condition interaction (HR: *F*_12, 132_ = 91.04, *P* < 0.01, *η*_p_^2^ = 0.89; MAP: *F*_12, 132_ = 10.40, *P* < 0.01, *η*_p_^2^ = 0.49). Follow-up *post-hoc* comparisons for set × condition interaction indicated that the HR and MAP values were unchanged during CON. In contrast, the HR values during the exercise session were significantly increased in LRE and HRE. The HR responses were significantly greater in HRE than in LRE. The MAP values during the exercise session were significantly increased during HRE but not during LRE. In addition, the values of RPE during the exercise session were gradually increased during LRE and HRE; they were greater during HRE than during LRE (set: *F*_1.56, 17.11_ = 24.24, *P* < 0.01, *η*_p_^2^ = 0.67; condition: *F*_1, 11_ = 60.00, *P* < 0.01, *η*_p_^2^ = 0.85; set × condition interaction: *F*_2.47, 27.13_ = 5.00, *P* < 0.01, *η*_p_^2^ = 0.31) (data not shown).

**Table 2:** Changes in cardiovascular responses during exercise.

	Pre	1^st^ set	2^nd^ set	3^rd^ set	4^th^ set	5^th^ set	6^th^ set
***HR (bpm)***							
**CON**	65.8 ± 8.5	67.0 ± 9.1	65.5 ± 8.0	66.0 ± 8.7	65.6 ± 8.2	64.3 ± 7.5	65.6 ± 8.4
**LRE**	66.6 ± 8.9	98.3 ± 11.2 ^a^*	99.2 ± 10.2 ^a^*	98.7 ± 8.9 ^a^*	99.6 ± 8.5 ^a^*	101.0 ± 9.4 ^a^*	104.8 ± 10.3 ^a^^c^^d^^e^*
**HRE**	66.9 ± 8.2	122.4 ± 15.5 ^a^*^#^	127.7 ± 12.6 ^a^*^#^	130.9 ± 12.8 ^a^*^#^	135.2 ± 15.8 ^a^^b^*^#^	137.9 ± 16.6 ^a^^b^*^#^	141.8 ± 16.0 ^a^^b^^c^^d^*^#^
***MAP (mmHg)***							
**CON**	88.5 ± 6.1	87.4 ± 6.5	86.9 ± 6.6	86.8 ± 7.7	86.6 ± 6.4	87.2 ± 7.8	88.6 ± 8.5
**LRE**	86.4 ± 7.6	88.9 ± 7.1	88.8 ± 6.3	89.1 ± 7.1	89.6 ± 7.8	89.7 ± 7.1	90.2 ± 6.8
**HRE**	87.6 ± 7.0	93.0 ± 8.0 *	95.9 ± 7.6 ^a^*^#^	98.3 ± 6.7 ^a^*^#^	98.7 ± 6.6 ^a^*^#^	99.6 ± 7.2 ^a^^b^*^#^	100.1 ± 6.8 ^a^^b^*^#^

Values are presented as the means ± SD. HR; heart rate, MAP; mean arterial pressure.

^a^*P* < 0.05 vs. Pre,

^b^*P* < 0.05 vs. 1^st^ set,

^c^*P* < 0.05 vs. 2^nd^ set,

^d^*P* < 0.05 vs. 3^rd^ set,

^e^*P* < 0.05 vs. 5^th^ set,

*P < 0.05 vs. corresponding CON condition,

^#^*P* < 0.05 vs. corresponding LRE condition.

The results of EMG activity of the quadriceps muscle and NIRS-derived deoxy-Hb/Mb during the 2 resistance exercise conditions are presented in Table 3. There were significant main effects for condition (iEMG (VL): *F*_1, 11_ = 73.98, *P* < 0.01, *η*_p_^2^ = 0.87; iEMG (VM): *F*_1, 11_ = 17.20, *P* < 0.01, *η*_p_^2^ = 0.61; iEMG (RF): *F*_1, 11_ = 78.08, *P* < 0.01, *η*_p_^2^ = 0.88; deoxy-Hb/Mb: *F*_1, 11_ = 8.94, *P* < 0.01, *η*_p_^2^ = 0.45) and set × condition interaction (iEMG (VL): *F*_5, 55_ = 3.24, *P* < 0.05, *η*_p_^2^ = 0.23; iEMG (VM): *F*_5, 55_ = 4.10, *P* < 0.01, *η*_p_^2^ = 0.27; deoxy-Hb/Mb: *F*_2.16, 23.78_ = 11.61, *P* < 0.01, *η*_p_^2^ = 0.51). However, there were no significant main effects for set (iEMG (VL): *F*_1.52, 16.71_ = 2.74, *P* > 0.05, *η*_p_^2^ = 0.20; iEMG (VM): *F*_1.47, 16.13_ = 0.59, *P* > 0.05, *η*_p_^2^ = 0.05; iEMG (RF): *F*_1.43, 15.69_ = 1.83, *P* > 0.05, *η*_p_^2^ = 0.14; deoxy-Hb/Mb: *F*_1.28, 14.11_ = 0.70, *P* > 0.05, *η*_p_^2^ = 0.06) and set × condition interaction (iEMG (RF): *F*_1.85, 20.32_ = 0.67, *P* > 0.05, *η*_p_^2^ = 0.06). Follow-up *post-hoc* comparisons for set × condition interaction indicated that the EMG activities of the quadriceps VL and VM muscles during resistance exercise session were significantly greater during HRE than during LRE. Similarly, the increases in deoxy-Hb/Mb of quadriceps VL muscle during the exercise session were significantly greater during HRE than during LRE.

**Table 3:** Changes in skeletal muscle responses during exercise.

	1^st^ set	2^nd^ set	3^rd^ set	4^th^ set	5^th^ set	6^th^ set
***iEMG (%MVC)***						
* Vastus lateralis*						
* ***LRE**	72.2 ± 9.0	69.9 ± 8.5	70.5 ± 7.9	70.5 ± 8.6	72.0 ± 9.5	71.8 ± 9.1
* ***HRE**	89.7 ± 3.4 ^#^	89.9 ± 3.5 ^#^	91.0 ± 4.3 ^#^	92.0 ± 5.9 ^#^	92.9 ± 6.0 ^#^	93.4 ± 5.6 ^a^^#^
* Vastus medialis*						
* ***LRE**	70.1 ± 15.6	68.1 ± 14.8	68.0 ± 13.7	67.8 ± 13.4	69.1 ± 12.5	68.8 ± 13.2
* ***HRE**	91.4 ± 15.2 ^#^	92.7 ± 15.3 ^#^	93.5 ± 15.3 ^#^	92.7 ± 15.4 ^#^	94.0 ± 15.9 ^#^	93.8 ± 15.0 ^#^
* Rectus femoris*						
* ***LRE**	58.6 ± 11.8	55.7 ± 9.7	56.1 ± 10.5	56.8 ± 10.9	58.3 ± 9.8	57.4 ± 10.5
* ***HRE** ^#^	89.3 ± 8.1	88.8 ± 8.8	88.6 ± 9.1	89.8 ± 10.4	90.0 ± 10.0	90.6 ± 10.6
***Deoxy-Hb/Mb (%ischemia)***						
* ***LRE**	69.7 ± 23.7	68.0 ± 22.7	67.4 ± 21.8	66.1 ± 21.3	64.6 ± 20.8	62.9 ± 19.5
* ***HRE**	75.1 ± 27.4	80.0 ± 26.4 ^#^	80.8 ± 25.6 ^#^	80.4 ± 26.5 ^#^	81.2 ± 25.5 ^#^	80.6 ± 24.1 ^#^

Values are presented as the means ± SD. iEMG; integration of the rectified electromyographic, MVC; maximal voluntary contractions, Hb; hemoglobin, Mb; myoglobin.

^a^*P* < 0.05 vs. 1^st^ set;

^#^*P* < 0.05 vs. LRE.

### CWST-measured IC

The reaction times and response accuracies for neutral and incongruent trials of the CWST during three conditions are shown in Table 4. The reaction times and response accuracies did not differ significantly between the baseline and pre-intervention data in all three conditions. With regard to response accuracy, there were no significant main effects or interactions for any factor. In contrast, with regard to reaction time, there were significant main effects for type of CWST (*F*_1, 11_ = 54.90, *P* < 0.01, *η*_p_^2^ = 0.83), pre/post (*F*_1, 11_ = 99.36, *P* < 0.01, *η*_p_^2^ = 0.90), condition (*F*_2, 22_ = 4.13, *P* < 0.05, *η*_p_^2^ = 0.27), and type of CWST × pre/post × condition interaction (*F*_2,22_ = 19.34, *P* < 0.01, *η*_p_^2^ = 0.64). Follow-up *post-hoc* comparisons for type of CWST × pre/post × condition interaction indicated that the reaction time of the neutral trial was significantly shorter than that with incongruent trial. The reaction time of neutral and incongruent trials in the post-intervention data was significantly shortened by LRE and HRE, but not by CON. The reaction time of the neutral trial with HRE was significantly shorter than that with CON. In an incongruent trial, the reaction time during both exercise conditions was significantly shorter than the time during the CON condition, and the time during HRE was significantly shorter than the time during LRE.

**Table 4:** Changes in reaction time and response accuracy at the CWST.

	Reaction time (msec)		Response accuracy (%)	
	Pre	Post	Pre	Post
***Neutral task***				
**CON**	10805 ± 2044	10876 ± 1917	98.7 ± 1.9	97.9 ± 2.9
**LRE**	10758 ± 1626	10264 ± 1482 ^a^	97.3 ± 2.0	98.1 ± 1.9
**HRE**	10955 ± 1617	9835 ± 1296 ^a^*	98.4 ± 1.9	98.5 ± 2.2
***Incongruent task***				
**CON**	12062 ± 2038 ^†^	12283 ± 2107 ^†^	98.3 ± 1.7	98.8 ± 1.0
**LRE**	11990 ± 1844 ^†^	10985 ± 1606 ^†^^a^*	97.6 ± 2.0	97.6 ± 2.0
**HRE**	12258 ± 2010 ^†^	10233 ± 1319 ^†^^a^*^#^	97.7 ± 2.0	97.9 ± 3.2

Values are presented as means ± SD.

^†^*P* < 0.05 vs. incongruent task;

^a^*P* < 0.05 vs. Pre;

*P < 0.05 vs. CON;

^#^*P* < 0.05 vs. LRE.

**Fig 2:**
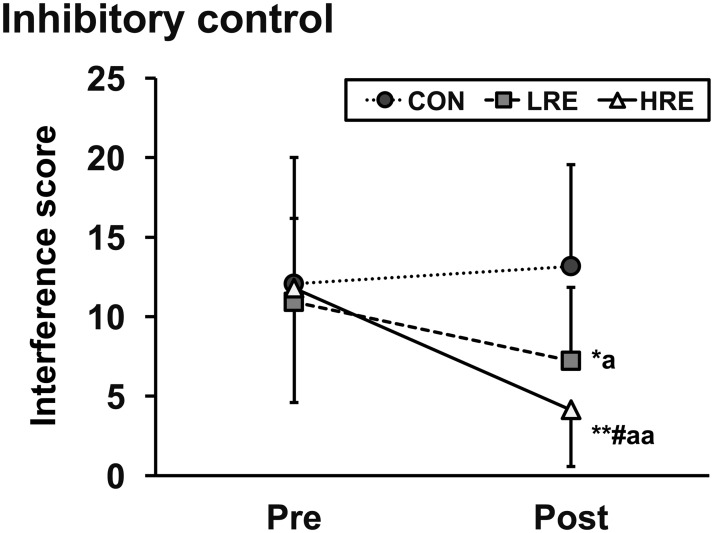
Changes in reverse-Stroop interference score of CWST. A panel illustrates the changes in reverse-Stroop interference score at the pre- and post-CWST periods. The values are shown as the mean ± SD. *,** vs. CON *P* < 0.05, 0.01; ^#^ vs. LRE *P* < 0.05; ^a,aa^ vs. Pre *P* < 0.05, 0.01.

The changes in the reverse-Stroop interference score of the CWST in all conditions are presented in Fig 2. A reverse-Stroop interference score did not differ significantly between the baseline and pre-intervention data in all three conditions (11.4 ± 6.2 vs. 12.1 ± 7.9 for CON; 10.7 ± 6.2 vs. 10.9 ± 5.3 for LRE; 11.8 ± 7.5 vs. 11.8 ± 7.2 for HRE). There were significant main effects for pre/post (*F*_1, 11_ = 16.17, *P* < 0.01, *η*_p_^2^ = 0.60), condition (*F*_1.37, 15.03_ = 4.26, *P* < 0.05, *η*_p_^2^ = 0.28), and pre/post × condition interaction (*F*_2, 22_ = 17.60, *P* < 0.01, *η*_p_^2^ = 0.62). Importantly, follow-up *post-hoc* comparisons for pre/post × condition interaction indicated that the reverse-Stroop interference scores immediately after LRE and HRE were significantly reduced compared to those before exercise (*P* < 0.05, Cohen’s *d* = 0.75; *P* < 0.01, Cohen’s *d* = 1.35), but not CON (*P* > 0.05, Cohen’s *d* = 0.15). In addition, the reverse-Stroop interference score immediately after both exercises were significantly greater than those during CON (CON vs. LRE: *P* < 0.05, Cohen's *d* = 1.06; CON vs. HRE: *P* < 0.01, Cohen's *d* = 1.74). Moreover, the score immediately after HRE was significantly greater than the score immediately after LRE (*P* < 0.05, Cohen's *d* = 0.75).

**Fig 3:**
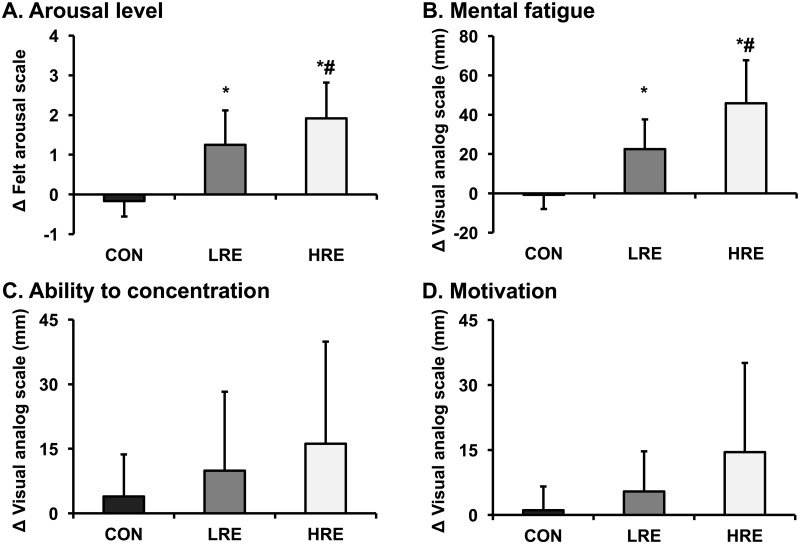
Changes in psychological parameters for CWST. The panels illustrate the changes in arousal level (A), mental fatigue (B), ability to concentrate (C), and motivation (D) in the pre- and post-CWST periods among all conditions. The arousal level was evaluated by felt arousal scale. Mental fatigue, ability to concentrate, and motivation was evaluated by visual analog scale. The values are shown as the mean ± SD. * vs. CON *P* < 0.05, ^#^ vs. LRE *P* < 0.05.

The changes in the psychological parameters for CWST between pre- and post-exercise are presented in Fig 3. There were significant main effects for condition (FAS: *F*_2, 22_ = 33.80, *P* < 0.01, *η*_p_^2^ = 0.76; Fatigue: *F*_2, 22_ = 32.15, *P* < 0.01, *η*_p_^2^ = 0.75). However, there were no significant main effects for condition (Concentration: *F*_2, 22_ = 1.91, *P* > 0.05, *η*_p_^2^ = 0.15; Motivation: *F*_1.24, 13.58_ = 3.41, *P* > 0.05, *η*_p_^2^ = 0.24). Follow-up *post-hoc* comparisons indicated that the changes in FAS-derived arousal levels in both resistance exercise conditions were significantly greater than the changes during the CON conditions (CON vs. LRE: *P* < 0.01, Cohen's *d* = 2.11; CON vs. HRE: *P* < 0.01, Cohen's *d* = 3.00), and the level during HRE was significantly greater than the level during LRE (*P* < 0.05, Cohen's *d* = 0.75). Similarly, the changes in VAS-derived mental fatigue during LRE showed a similar result (CON vs. LRE: *P* < 0.01, Cohen's *d* = 1.96; CON vs. HRE: *P* < 0.01, Cohen's *d* = 2.86; LRE vs. HRE: *P* < 0.01, Cohen's *d* = 1.24).

## Discussion

In the present study, IC at the baseline was equal among the three conditions. Moreover, IC before experimental condition was not different from that of the baseline in all three conditions. Furthermore, IC did not differ before and immediately after the CON was implemented. These results clearly suggest that subjects who participated in the present study had no learning effect for CWST. Thus, the major finding of the present study was that, without learning effect, an acute bout of localized resistance exercise using knee extension alone improved IC immediately after both HRE and LRE. In addition, we showed that this IC improvement was greater following HRE than following LRE, as associated with enhancements in cardiovascular, skeletal muscle, and psychological responses. Previous studies have reported that whole-body resistance exercise with multiple exercise events could improve EF, including IC, immediately after exercise and during post-exercise recovery [16,17,36]. Thus, the present findings further extend the effectiveness of resistance exercise for EF by showing that even localized resistance exercise using only a single exercise event could improve IC.

During exercise sessions, whole-body resistance exercise is performed by recruiting a large number of muscle groups because it is programmed by combining upper and lower body exercises, including multi-joint exercise (e.g., leg press and bench press, etc) [16,17,36]. In addition, whole-body resistance exercise necessarily consists of relatively large numbers of sets in an exercise session, because it is performed using multiple events [16,17]. In a previous study, Chang and Etnier (2009) reported that whole-body resistance exercise, which consisted of 12 sets, 6 exercises events with 2 sets per event, improved IC as evaluated using the Stroop test [16]. Moreover, Pontifex et al. (2009) reported that whole-body resistance exercise, which consisted of 21 sets, 7 exercise events with 3 sets per event, improved cognitive performance as evaluated using a modified Sternberg working memory task [36]. In contrast, localized resistance exercise in the present study consisted of 6 sets using only a knee extension exercise. Thus, localized resistance exercise is performed by recruiting a limited number of muscle groups, and consisted of relatively small numbers of sets in an exercise session, suggesting that various local resistance exercise-induced stimulations, including mechanical, neuromuscular, and metabolic stresses, are naturally low compared with the stimulations induced by whole-body resistance exercise [26].

Due to the relatively large number of sets, whole-body resistance exercise requires a relatively long exercise duration, which suggests that there may be a relatively high aerobic demand during an exercise session, similar to the effect of aerobic exercise on EF. In fact, previous studies have determined that long-term whole-body resistance exercise could increase aerobic capacity, such as maximum oxygen uptake [37,38]. Previous studies have examined the acute effect on EF using whole-body resistance exercise with exercise duration ranging from 30 to 45 min [16,36] However, the duration of the localized resistance exercise in the present study was relatively shorter being 17 min, suggesting that localized resistance exercise has a relatively lower aerobic demand during an exercise session. Therefore, compared to whole-resistance exercise, although localized resistance exercise results in various stimulations which seem insufficient for IC improvement, it may be sufficient for improving IC.

Previous studies have suggested that the aerobic exercise-induced IC improvement might be related to enhanced cerebral neural activity and arousal [8–10]. In accordance with aerobic exercise responses, resistance exercises may also enhance neural activity in the brain, which is associated with enhancements of cardiovascular, skeletal muscle, and psychological responses [22,23,39,40]. For example, Sander et al. (2010) reported that increased cardiovascular responses, such as HR and MAP, were associated with enhanced cerebral neural activity during an isometric handgrip exercise [39]. Moreover, several previous studies reported that increased skeletal muscle responses, such as increased EMG activity and decreased tissue oxygenation, were associated with enhanced cerebral neural activity during several types of resistance exercise [22,23] Furthermore, Berchicci et al. (2013) reported that increased psychological responses, such as RPE, were associated with enhanced cerebral neural activity during isometric knee extension exercise [40]. The present findings showed that localized resistance exercise-induced enhancements in cardiovascular, skeletal muscle, and psychological responses were greater following HRE than following LRE. Of those, arousal level immediately after exercise was greater in HRE than in LRE, indicating that this may at least partially explain the differences in the magnitude of IC improvement between HRE and LRE, associated with a cognitive-energetic model [41]. Thus, compared to LRE and CON, greater improvement in IC immediately after HRE may be explained by greater cerebral neural activation, which is related to the increases in several physiological and psychological response levels. Taken together, the findings suggest that when localized resistance exercise performed, HRE may be more effective in improving IC than LRE.

Several previous studies examined the dose-response effect of whole-body resistance exercise on EF improvement [16,17]. Chang et al. (2009) reported that Stroop task-measured IC improvement immediately after whole-body resistance exercise was greater when it was of moderate-intensity rather than of low-intensity or high-intensity, and was comparable between low-intensity and high-intensity exercises [16]. However, the present study demonstrated that resistance exercise-induced improvement in IC was greater immediately after HRE than immediately after LRE. Although a reasonable explanation for this discrepancy is unavailable, compared with whole-body resistance exercise, localized resistance exercise may have relatively low values for other exercise components, such as exercise duration, exercise volume, and exercised muscle groups. Thus, further study is needed to examine the contributions of various exercise components in order to determine an optimal resistance exercise protocol for effectively improving EF in the clinical setting.

One possible limitation of the present study is that we proposed that the acute resistance exercise-induced increases in cardiovascular, skeletal muscle, and psychological responses may play important roles in IC improvement, potentially by increasing neural activity in the brain. Nevertheless, we could not provide direct evidence linking physiological and psychological responses, IC improvement, and cerebral neural activation because we did not measure the cerebral neural activation during the two resistance exercise conditions, which could be measured using functional magnetic resonance imaging, functional near-infrared spectroscopy, and electroencephalography [42]. Thus, further studies are needed to determine the regulator(s) of resistance exercise-induced IC improvement by incorporating the measurement of cerebral neural activation.

Another limitation was that the present study did not examine the impact of moderate-intensity localized resistance exercise on IC. Previous studies have demonstrated that EF improvement induced by whole-body resistance exercise is greater when it is of moderate-intensity rather than of low-intensity or high-intensity. With regard to whole-body resistance exercise, it has been suggested that there may be an inverted-U relationship between exercise intensity and IC improvement. Moreover, although we determined that localized resistance exercise-induced IC improvement was greater in HRE than in LRE, the present study did not evaluate differences in exercise volume due to the same repetitions between HRE and LRE, which may be naturally related to improving IC by enhancing cardiovascular, skeletal muscle, and psychological responses. Furthermore, we did not examine the impact of localized resistance exercise on IC improvement during post-exercise recovery, which would provide information related to the endurance of the exercise-induced IC improvements [11,13–15]. Therefore, further studies are needed to examine the effect of moderate-intensity exercise and exercise volume on IC improvement during post-exercise recovery in order to establish an effective resistance exercise prescription for improving EF.

## Conclusion

The present findings showed that an acute bout of localized resistance exercise could improve IC immediately after exercise. Moreover, the localized resistance exercise-induced improvement in IC was greater following HRE than following LRE, and was associated with enhancements in cardiovascular, skeletal muscle and psychological responses. Therefore, we propose that, compared to whole-body resistance exercise and aerobic exercise, localized resistance exercise that is performed by recruiting limited muscle groups may be sufficient for improving IC.
